# Dietary Quality Among Older Overweight or Obese Veterans With Dysmobility

**DOI:** 10.1093/geroni/igaa057.743

**Published:** 2020-12-16

**Authors:** Elizabeth Parker, Lauren Bergamo, Will Perez, Leslie Katzel, Alice Ryan, Steven Prior, Monica Serra, Odessa Addison

**Affiliations:** 1 University of Maryland School of Medicine, Baltimore, Maryland, United States; 2 Baltimore VA Medical Center, Baltimore, Maryland, United States; 3 University of Maryland School of Public Health, College Park, Maryland, United States; 4 UTHSCSA, San Antonio, Texas, United States; 5 University of Maryland School of Medicine, Glen burnie, Maryland, United States

## Abstract

Older adults have unique dietary challenges due to a myriad of factors including age-related taste and smell changes and lack of nutrition knowledge that increase the risk for poor dietary quality. Healthier dietary quality is associated with higher muscle mass, strength and physical performance which may reduce the development of frailty and disability later in life; however, few studies have examined dietary quality among older Veterans with limited physical functioning. The purpose of this study was to examine overall dietary quality among older, overweight/obese veterans with dysmobility. Habitual dietary intake was assessed at baseline using three, nonconsecutive 24-hour recalls and used to calculate healthy eating index (HEI-2015; higher scores indicate higher diet quality). Twenty-eight participants were included in analysis: 93% male; 54% black; aged=69.5±7.0 years; BMI=35.5±5.4 kg/m2. Means and standard deviations were calculated for average intake of total daily energy (2184±645 kcals) and protein (0.89±0.3g/kg), daily servings of fruits (0.84±0.94) and vegetables (1.3±0.87), and HEI-2015 (52.8±13.4). Overall, 96% consumed fewer than the recommended 5 daily servings of fruits and vegetables and 68% consumed <1.0g/kg/d of protein (1.0-1.3g/kg/d recommended for older adults). Mean HEI-2015 was below the US national average for adults >65 years (2015-2016 NHANES 65+ years: 64.0), suggesting poor dietary quality among our sample. This pilot study suggests that dietary intake quality is suboptimal in older, obese Veterans with disability and highlights the need to identify strategies that improve dietary intake quality of older Veterans who may benefit from obesity and disability management.

